# Developing scalable woven textile-based scaffolds for tubular tissues with Core–Sheath nanofibre yarns and tuneable weave architectures

**DOI:** 10.3389/fbioe.2025.1655852

**Published:** 2025-11-20

**Authors:** Anna Doersam, Luis Larrea Murillo, Celina Jones, Olga Tsigkou

**Affiliations:** 1 Department Materials, Henry Royce Institute, University of Manchester, Manchester, United Kingdom; 2 Department Materials, University of Manchester, Manchester, United Kingdom; 3 Manchester Fashion Institute, Manchester Metropolitan University, Manchester, United Kingdom

**Keywords:** tissue engineering, textile technology, tubular tissues, textile-based scaffolds, nanofiber-based techniques, electrospinning

## Abstract

**Introduction:**

Engineering functional tubular tissues requires scaffolds that replicate the anatomical complexity, mechanical behaviour, and biological microenvironment of native organs.

**Methods:**

Here, we present a scalable, automated weaving strategy combining PLA multifilament cores with electrospun PCL nanofibre sheaths to fabricate biomimetic core-sheath yarns. These yarns were woven into tubular scaffolds with tunable architectures, enabling precise control over surface topography, porosity, and mechanical compliance.

**Results:**

While microscale cues such as fibre diameter and chemistry are critical, we show that mesoscale weave geometry also modulates cell spatial distribution, orientation, and network formation. Plain weave patterns supported uniform endothelial and smooth muscle cell attachment, viability, and proliferation, while more complex weaves modulated cytoskeletal organisation and multilayer formation. Mechanical characterisation confirmed enhanced strength and elasticity compared to pure nanofibre yarns, yielding more tissue-like mechanical performance.

**Discussion/Conclusion:**

This approach overcomes key limitations of traditional electrospun membranes and manual weaving methods by offering reproducibility, structural stability, and design flexibility. Our results demonstrate that controlling yarn morphology and mesoscopic weave architecture can guide cell behaviour and tissue organisation, providing a promising platform for engineering vascular, tracheal, and oesophageal grafts with clinically relevant properties.

## Introduction

The failure or dysfunction of tubular tissues such as blood vessels, trachea, and oesophagus, due to trauma, disease, or congenital defects, presents significant clinical challenges for a variety of associated medical conditions. Cardiovascular diseases alone accounts for approximately 17.9 million deaths annually worldwide, underscoring the urgent need for effective regenerative solutions (Lindstrom et al., 2022). Tracheal injuries, often resulting from prolonged intubation or trauma, oesophageal disorders, including congenital anomalies and cancers, also represent substantial healthcare burdens, demanding improved therapeutic strategies. Current clinical solutions such as synthetic grafts, autologous transplants, and decellularized scaffolds have been essential in clinical practice. Nonetheless, they encounter limitations including mechanical mismatch, limited regenerative capacity, susceptibility to infection, and lack of functional integration with host tissue (Lindstrom et al., 2022; Pennel and Zilla, 2020; Mattioli et al., 2021). These limitations often result in disorganized extracellular matrix (ECM) deposition, impaired cellular alignment, and compromised functional integration, leading to high revision rates and poor long-term clinical outcomes, while failures are further driven by mechanical mismatch, infection, and limited durability of synthetic grafts, while even advanced bioreactor approaches still face challenges in achieving regeneration and long-term functional integration (de Valence et al., 2012; Szafron et al., 2019; Sarkar et al., 2023).

Tissue-engineered approaches offer significant promise for overcoming these limitations by providing scaffolds capable of actively supporting and guiding tissue regeneration (Lanza et al., 2020). However, the successful regeneration of tubular tissues requires scaffolds that not only replicate the anatomical geometry and hierarchical organization of native tissues but also match their biomechanical properties and biological functionality (Cao et al., 2023). Current fabrication techniques, including electrospinning have demonstrated potential in providing nanoscale ECM mimicry and complex structures (Xu et al., 2004; Ingavle and Leach, 2014). However, electrospun scaffolds often lack the mechanical compliance and dimensional stability required for load-bearing tubular applications (Abdal-hay et al., 2018; Lee et al., 2007).

Textile-based scaffolds, particularly woven constructs, offer compelling alternatives due to their inherent mechanical strength, dimensional stability, controlled porosity, and reproducible geometry (Seeram, 2001; Doersam et al., 2022; Jiao et al., 2020; Jiang et al., 2021; Akbari et al., 2016). The weaving process involves the precise interlacing of warp and weft yarns, facilitating the creation of seamless tubular structures through modified shuttle looms or circular looms. Such seamless construction eliminates weak points or seams that result in mechanical failure, leak sites, or areas susceptible to uneven stress distribution (Joseph et al., 2018). Commercially available woven grafts, currently used in vascular surgery, such as polyethylene terephthalate (PET, commercially known as Dacron®) and expanded polytetrafluoroethylene (ePTFE), have demonstrated clinical success (Spadaccio et al., 2019). Nonetheless, although these bioinert textile grafts are biocompatible and effectively restore critical functions, they primarily act as passive structures that do not actively interact with biological systems (Ratner, 2023; Spadaccio et al., 2019; Baba et al., 2022). Their long-term performance is limited by issues such as mechanical mismatch with native tissues and lack of biological integration. Advances like drug-eluting grafts aim to mitigate these issues by gradually releasing bioactive agents to enhance integration and reduce complications (Domb and Khan, 2014; Zhang et al., 2019). The Gore® PROPATEN® Vascular Graft by W.L. Gore & Associates, is coated with heparin, a bioactive agent that helps reduce thrombosis by inhibiting platelet activation (Baba et al., 2022). Despite their promise, bio-functionalised textile grafts lack the compliance and tissue ingrowth, leading to flow dynamic mismatches that impact performance (Baba et al., 2022).

Recent developments in incorporating nanofibres into woven textile scaffolds offer significant promise to address these limitations by providing essential nanoscale topographical cues essential for cell adhesion, proliferation, and differentiation (Xie et al., 2021; Aghaei-Ghareh-Bolagh et al., 2019; Wu Y. et al., 2017; Khil et al., 2005). For example, electrospun PLLA scaffolds with nanoscale fibrous topography have been shown to enhance focal adhesion and spreading of endothelial cells, with aligned fibres further promoting elongation and orientation compared to randomly oriented structures (Moffa et al., 2014). More recently, nanoporous electrospun fibres were found to improve adhesion of epithelial and endothelial cells, while smoother fibres supported the formation of tighter junctions in epithelial monolayers (Jain et al., 2023). These findings illustrate how subtle nanoscale variations can elicit distinct cell responses, underscoring the potential of nanofibre-textile hybrids to improve graft integration.

The majority of these efforts utilize manual weaving methods which severely limit scalability, reproducibility, and complexity required for practical clinical applications (Wu et al., 2016; Cai et al., 2023; Aghaei-Ghareh-Bolagh et al., 2019). Electrospinning technology, in particular conjugate electrospinning set-ups, has demonstrated substantial promise in producing continuous nanofibre yarns. Core-sheath nanofibre yarns uniquely integrate the mechanical robustness of a polymeric core with native ECM-like nanoscale architectural features in the sheath, which can enhance cellular interactions without sacrificing structural integrity. This approach allows for multi-scale biomimicry, significantly improving both biological functionality and mechanical compatibility (Wu S. et al., 2017; Shaohua et al., 2017).

Electrospinning technology, particularly when employed in core-sheath configurations, has emerged as an effective method to produce continuous nanofibre yarns (NYs) (Liu et al., 2019; Fakhrali et al., 2014; Zhou et al., 2010; Li et al., 2018; Li et al., 2022). In textile engineering, core-sheath yarns are defined as composite yarn structures in which one type of fibre (the core) is encapsulated by another fibre type (the sheath). Each distinct layer can be composed of different types of polymers to tune various yarn characteristics. The core typically provides mechanical strength, durability, elasticity, or structural integrity, while the sheath contributes desired surface characteristics, biological activity, or functional attributes. Specifically, in the context of tissue engineering, core-sheath yarns consist of an inner polymer core offering structural support, surrounded by an outer sheath of nanofibres that enhance biological activity by closely mimicking native ECM features.

Herein, biomimetic tubular scaffolds were developed using an automated weaving approach, integrating core-sheath nanofibre yarns to replicate anatomical geometry and provide tunable yarn- and weave-level mechanical and surface properties while supporting relevant biological interactions in tubular-tissue contexts. This scalable and precise approach addresses key limitations of electrospun membranes by enabling controlled fibre morphology and customisable weave architecture. Such tunability is essential for engineering tubular grafts that meet the topographical and mechanical demands of complex tissues, particularly large vessels like the aorta.

## Materials and methods

### Fabrication of PLA-PCL multifilament/nanofibre textile scaffolds

A polymer solution (10% w/v) was prepared by dissolving Poly(ε-caprolactone) (PCL; Sigma Aldrich, United Kingdom, Cat. No. 440744-500G) in a solvent mixture of dichloromethane (DCM; Sigma Aldrich, United Kingdom) and N,N-dimethylformamide (DMF; Fisher Scientific, United Kingdom) at a 7:3 volume ratio. The solution was stirred continuously for 24 h at room temperature until fully dissolved.

**FIGURE 1:**
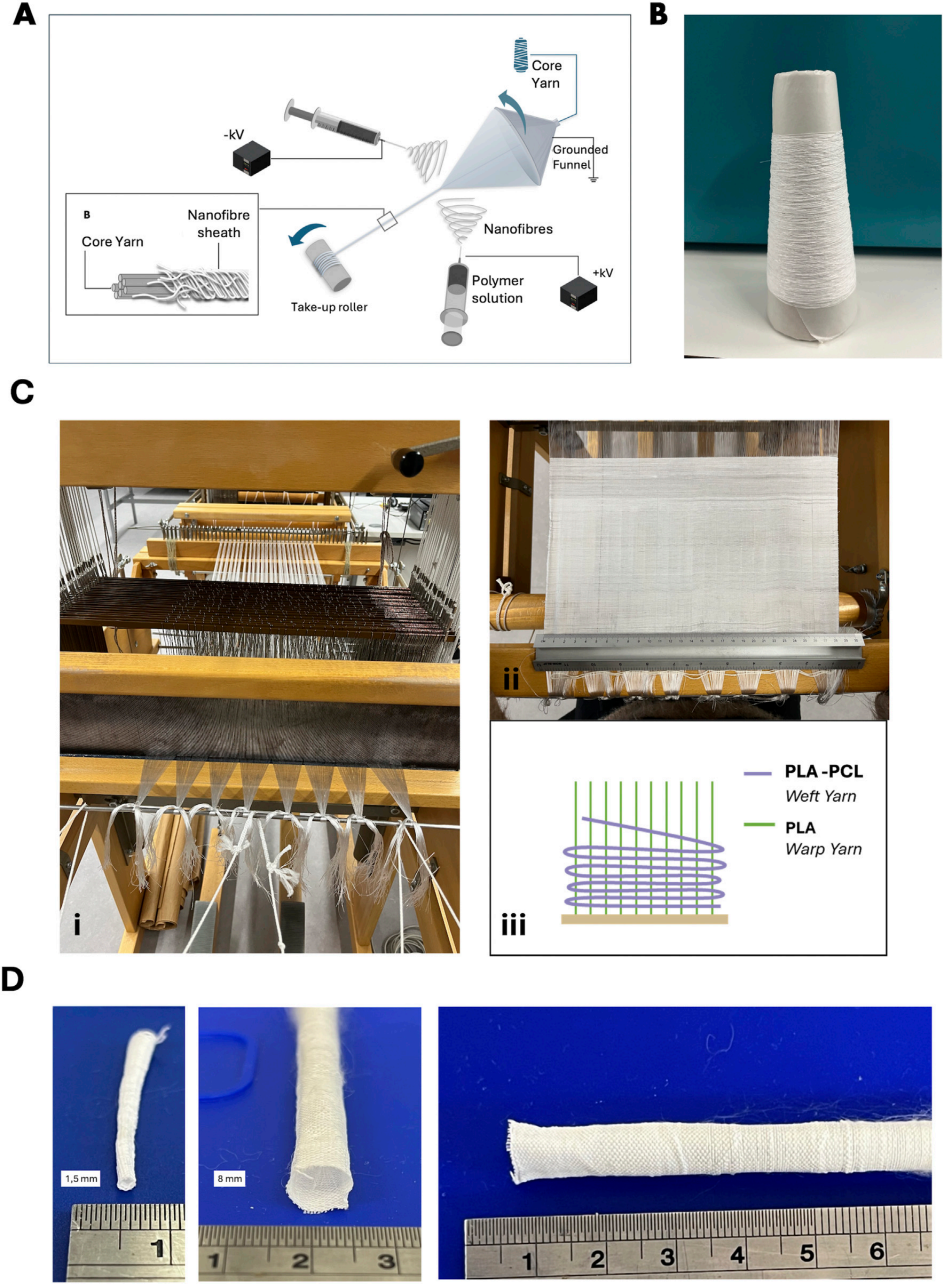
Fabrication of PLA-PCL nanofibre spacer fabric scaffolds. **(A)** Schematic illustration of the typical electrospinning system for fabricating nanofibre core-sheath yarn. **(B)** Photograph of a PLA-PCL nanofibre yarn package produced about 2 h. **(C)** Photographs of the weaving process (i) and PLA-PCL plain woven scaffolds (ii) Schematic illustration of yarn arrangements (iii), **(D)** Photographs of PLA-PCL plain woven tubes with 1.5 mm (i) and 8 mm diameter (ii).

Electrospinning was performed using a Fluidnatek® LE-500 electrospinning system (Bioinicia, Spain), equipped with dual syringe pumps, two oppositely charged 21G stainless steel needles, a rotating funnel collector, and an automated take-up reel (Figure 1A). The needles were placed 175 mm from the funnel collector, and voltages of −8 kV and +12 kV were applied to each needle, respectively. Polylactic acid (PLA) (NatureWorks, Ingeo Extend 4950D United States) multifilament yarn (warp-grade) was continuously fed through the centre of the rotating funnel, enabling electrospun nanofibres to deposit evenly around the PLA core, resulting in a continuous, stable core-sheath nanofibre yarn structure. Nanofibre yarns were collected onto the reel at a controlled winding speed between 14 and 18 rpm. A representative image of the PLA-PCL yarn package collected over a 2-h run is shown in Figure 1B.

Woven scaffold structures were fabricated using an Arm Patronic semi-automated loom (Figure 1Ci) (ARM AG CH-2507 Biglen) equipped with an electronic dobby mechanism for precise control over individual warp yarn movements (Figure 1Cii). PLA multifilament yarns were used as warp material, and the previously fabricated electrospun PLA-PCL core-sheath nanofibre yarns served as the weft material (Figure 1Ciii). Warp yarns and warp beams were prepared using a Hergeth Hollingsworth sample warper and subsequently threaded individually through the heald wires and frames. A reed density of 10 dents/cm was selected, and the warp yarns were threaded at a density of 2 ends/dent, resulting in a final warp density of 20 ends/cm. The weaving was performed at a constant warp tension to ensure uniform fabric quality. Multiple woven structures were produced to investigate how weave architecture influences scaffold properties. These structures included plain weave, 2 × 2 twill weave, sateen weave (R = 8, S = 5), broken twill (1/7 S with alternating 5 × 5 S-diagonal and 3 × 3 Z-diagonal), and shadow twill (warp repeat Rwp = 22, weft repeat Rwf = 3). While Figure 1D, shows a tubular scaffolds fabricated in a plain weave structure, consisting of PCL-PLA core–sheath yarns in the weft and PLA yarns in the warp, produced with customisable diameters.

### Material characterization

Morphological characteristics of core-sheath nanofibre yarns and woven scaffolds were examined using scanning electron microscopy (SEM; Tescan Vega3, United Kingdom) at 5 kV and a working distance of 12–25 mm. Samples were gold/palladium coated (10 nm thickness) prior to imaging. Fibre diameters and alignment were quantified using ImageJ from SEM images at ×500 magnification (n = 10). Yarn linear density (tex) was measured according to BS EN ISO 2060:1995 via the skein method. PLA-PCL core-sheath nanofibre yarns (10 m skeins) from five batches were weighed, averaged, and compared to reference yarns. Mechanical properties were determined on individual yarns to characterise tensile behaviour prior to scaffold fabrication, enabling determination of baseline yarn properties independent of scaffold architecture. Tests were performed using an Instron 5565 tensile tester (5 N load cell) following BS EN ISO 2062:2009 standards. Tensile tests were conducted at 250 mm/min crosshead speed, applying a pre-load of 0.5 cN/tex to determine yarn tenacity, with at least three replicates per yarn type. Further, tensile strength (σ) of the yarns was calculated using the measured tenacity and the known material density (ρ) values of polymers PLA (1.25 g/cm^3^) and PCL (1.145 g/cm^3^) using the following formula:

σ=Tenacity×ρ

The woven scaffold cover factor was calculated from warp and weft yarn densities and diameters. Surface roughness of scaffolds was analysed using a digital microscope (Keyence VHX-5000, United Kingdom) at ×10 magnification (n = 5). Height profiles were captured in warp and weft directions, calculating mean peak-to-valley heights (Rz). The woven scaffold cover factor was calculated from warp and weft yarn densities and diameters. Scaffold relative porosity was determined gravimetrically, measuring laser-cut samples (10 × 10 mm) and applying polymer density values to compute porosity.

### Surface wettability assessment

Water contact angles were evaluated with a Drop Shape Analyser (Krüss DSA100, Germany). A 1 µL water droplet was applied to scaffold surfaces (n = 5 per scaffold type), and angle changes were continuously recorded until absorption to assess scaffold hydrophilicity.

### Biocompatibility assessment of core-sheath nanofibre yarns and woven scaffolds

For biological evaluations, scaffolds were laser-cut into 10 × 10 mm samples, sterilised with ethanol (70%) and UV exposure (45 min/side), washed with PBS, and air-dried. Human umbilical vein endothelial cells (HUVECs) and human oesophageal smooth muscle cells (HOeSMCs) were used for biological evaluation. HUVECs were cultured in Endothelial Cell Growth Medium 2 (PromoCell, C-22011) supplemented with Supplement Mix (C-39216), L-glutamine, and 1% penicillin-streptomycin. HOeSMCs were maintained in high-glucose DMEM (Sigma-Aldrich, D5796) supplemented with 10% fetal bovine serum, 1% Antibiotic-Antimycotic, and L-glutamine. All cells were cultured at 37 °C in a humidified incubator with 5% CO_2_ and passaged at 80%–90% confluence. Dry scaffolds were seeded using a low-volume droplet method, 10 µL droplets. of cell suspension were evenly applied per scaffold, followed by a 2-h incubation at 37 °C to promote attachment. HUVECs were seeded at a density of 7.5 × 10^3^ cells/cm^2^, while HOeSMCs were seeded at 4 × 10^3^ cells/cm^2^. After the initial attachment phase, 500 µL of the appropriate complete medium was added to each well.

Cell viability was assessed at 24 h, 4 days, and 7 days using the LIVE/DEAD™ Viability/Cytotoxicity Kit (L3224, Life Technologies Ltd., United Kingdom), following the manufacturer’s instructions. Each scaffold was incubated in 300 µL of this solution for 30 min at 37 °C in the dark. After incubation, samples were gently washed with PBS to remove unbound dye and immediately imaged using a Leica SP8 confocal microscope (Leica Microsystems Ltd., Milton Keynes, United Kingdom). Samples were imaged using a Leica SP8 confocal microscope (Leica Microsystems Ltd., United Kingdom). Cell metabolic activity was quantified using a resazurin reduction assay (Resazurin sodium salt, R7017, Sigma-Aldrich, United Kingdom). A resazurin stock solution was prepared in PBS and diluted 1:10 (v/v) in complete culture medium immediately before use. Scaffolds were rinsed in once PBS, transferred to fresh wells containing 1 mL of the working solution, incubated for 3 h at 37 °C in a humidified incubator with 5% CO_2_. Following incubation, 200 µL of supernatant from each well was transferred to a black 96-well plate. Fluorescence was measured at 560 nm excitation and 590 nm emission using a FLUOROstar OPTIMA plate reader (BMG LabTech, Germany). Blank controls were used to correct background signal. Cell proliferation was assessed by total DNA content using the Quant-iT™ PicoGreen™ dsDNA Assay Kit (P7589, Thermo Fisher Scientific, United Kingdom), according to the manufacturer’s protocol. Samples were subjected to three freeze–thaw cycles, lysed in 1% Triton X-100 (X100, Sigma-Aldrich, United Kingdom) in TE buffer, and incubated for 3 h on a rocking platform at room temperature. Lysates were mixed with diluted PicoGreen reagent, and fluorescence was measured at 485 nm excitation and 520 nm emission using the FLUOROstar OPTIMA plate reader. DNA concentrations were calculated using a standard curve generated from known dsDNA standards.

### Statistical analysis

All statistical analyses were performed using GraphPad Prism (Version 10.4.2 (534), March 29, 2025) and Microsoft Excel 2025. For comparisons involving more than two groups, statistical significance was assessed by one-way ANOVA with Tukey’s multiple comparisons *post hoc* test. A *p*-value of <0.05 was considered statistically significant. Data are presented as mean ± standard error of the mean (SEM). Biological assays were conducted with two independent experiments; each performed in technical triplicates. Mechanical testing was performed on three independent yarn or scaffold samples per group. Morphological analyses (e.g., fibre diameter, pore size) were based on measurements from ten randomly selected SEM fields per sample across three independent samples.

## Results

### Morphological features of PLA-PCL core-sheath yarn

**FIGURE 2:**
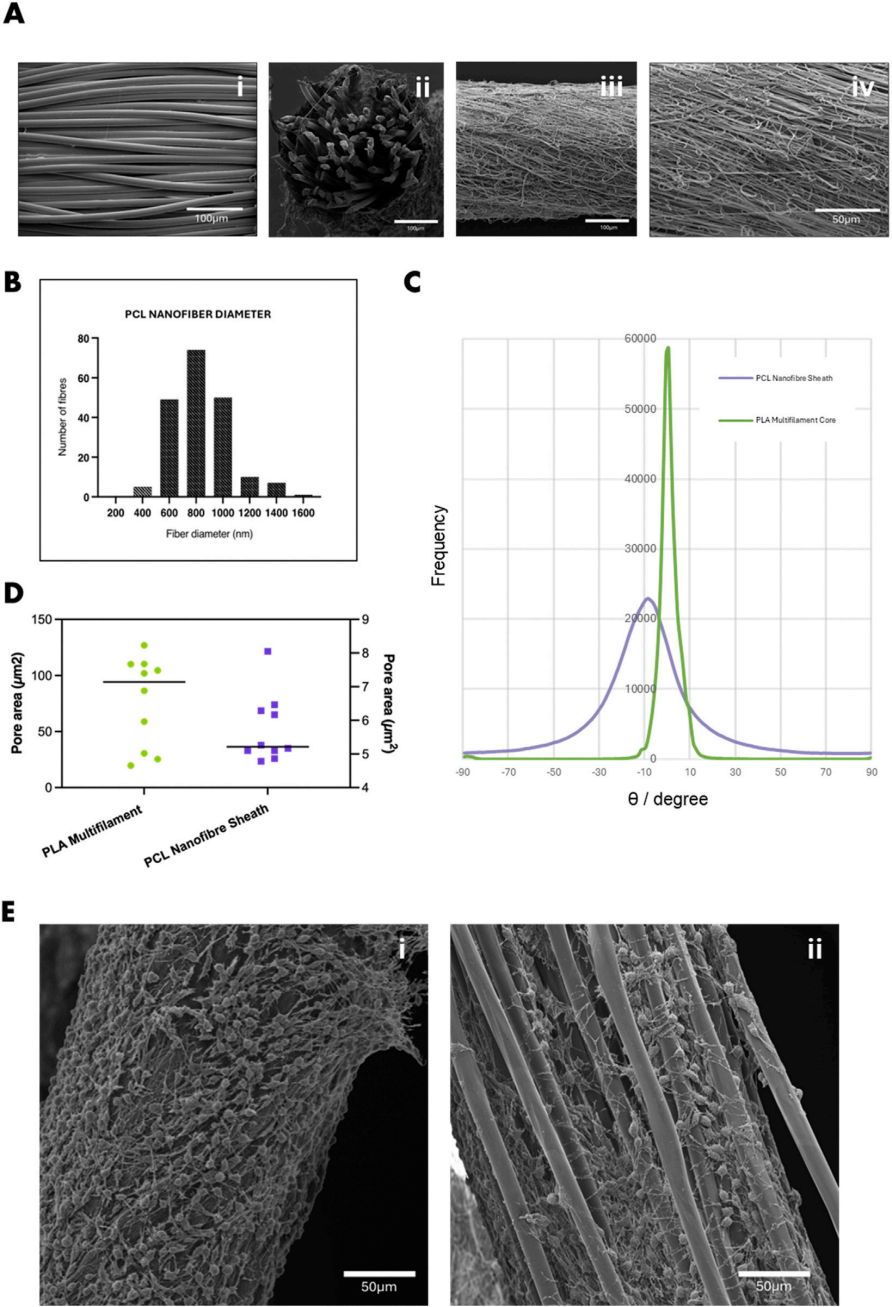
**(A)** SEM images showing (i) PLA multifilament core, (ii) cross-sectional view of the PLA–PCL core-sheath yarn, and (iii) surface morphology of the PCL nanofibre sheath (iv) magnified 1.5kx. **(B)** Histogram showing the diameter distribution of PCL nanofibres within the core-sheath yarn. **(C)** Quantification of fibre orientation relative to the yarn axis, indicating predominant alignment along the x-direction. **(D)** Average pore area within the PCL nanofibre sheath and PLA multifilament core, determined by SEM image analysis. **(E)** SEM images of 3T3 fibroblasts cultured for 7 days on (i) PCL nanofibre yarn, showing dense surface attachment and spreading, and (ii) PLA multifilament yarn, with cells infiltrating into inter-filament gaps. Scale bar = 50 µm.

The core-sheath yarn structure was successfully fabricated using conjugate electrospinning, combining a PLA multifilament core with an electrospun PCL nanofibre sheath. This approach produced a stable, continuous yarn with a well-defined architecture. SEM images confirmed the distinct morphological differences between the PLA core (Figures 2Ai,ii) and the PCL sheath (Figures 2Aiii,iv). The PLA multifilament core consisted of smooth, uniform filaments measuring 13 µm in diameter. In contrast, the electrospun PCL sheath comprised randomly distributed fibres with a mean diameter of 851.78 ± 253.23 nm (Figure 2B), within the biomimetic range known to support cellular adhesion and proliferation, particularly in vascular tissue engineering, tendon repair, and ECM-mimetic scaffolds (Mo et al., 2004; Han et al., 2019; Flavia et al., 2022; Shaohua et al., 2017; Qi et al., 2023). Quantitative analysis of scaffold porosity was conducted using particle analysis in FIJI (ImageJ) by applying threshold-based segmentation to SEM images. Analysis showed that the PCL nanofibre sheath had a mean pore area of 5.73 ± 0.69 µm^2^ and a surface porosity of 24.1% ± 1.27%. In contrast, the PLA multifilament core exhibited significantly larger inter-filament pore areas (94.19 ± 40.25 µm^2^) and a lower surface porosity of 9.92% ± 0.59% (Figure 2D). These fibres exhibit axial alignment (∼10°) relative to the multifilament core (Figure 2C), contributing to a uniform sheath layer approximately 9.98 ± 4.30 µm thick. Quantitative analysis of scaffold porosity was conducted using particle analysis in FIJI (ImageJ) by applying threshold-based segmentation to SEM images. Analysis showed that the PCL nanofibre sheath had a mean pore area of 5.73 ± 0.69 µm^2^ and a surface porosity of 24.1% ± 1.27%. In contrast, the PLA multifilament core exhibited significantly larger inter-filament pore areas (94.19 ± 40.25 µm^2^) and a lower surface porosity of 9.92% ± 0.59% (Figure 2D). To assess their suitability of tissue engineering applications, preliminary biocompatibility assessment of the PLA-PCL scaffolds using 3T3 fibroblasts was conducted. Differences in fibre diameter and pore area influenced seeded 3T3 fibroblast behaviour on the two yarn types (Figure 2E). On the electrospun PCL nanofibre sheath, the smaller fibre diameters and pore sizes promoted surface-level spreading and cytoplasmic extension (Figure 2Ei). In contrast, the larger diameter PLA multifilament yarns and wider inter-filament pores supported cell alignment along individual filaments and facilitated infiltration into the yarn interior (Figure 2Eii), suggesting distinct features of scaffold interaction based on architectural fibre structure.

### Mechanical characterization of PLA-PCL core-sheath yarn

**FIGURE 3:**
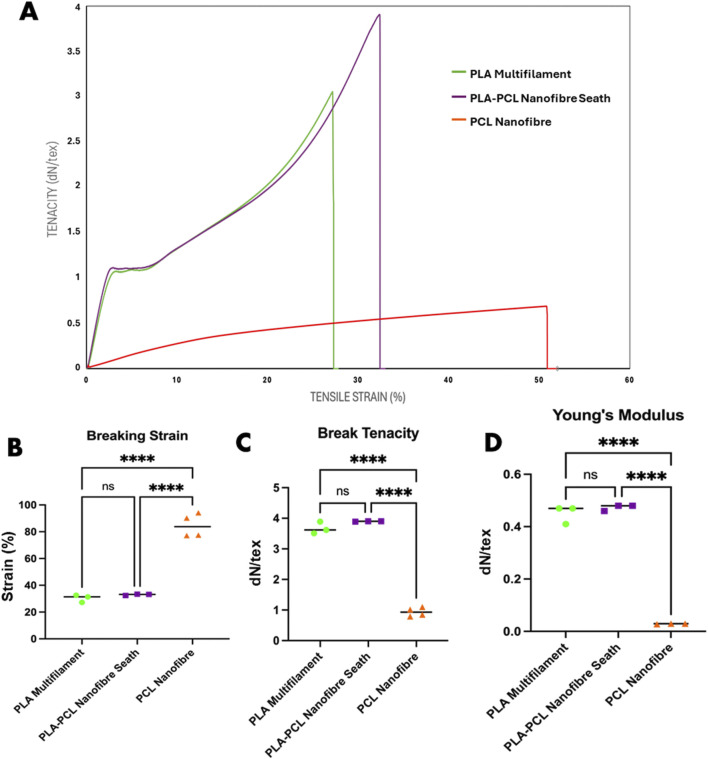
Mechanical analysis of PLA multifilament, PLA–PCL nanofibre sheath and PCL nanofibre yarns. **(A)** Stress/strain curves representative of mechanical properties of each yarn condition. **(B)** Mechanical property differences in breaking strain, **(C)** breaking tenacity, and **(D)** Young’s modulus. Statistical analysis: two-way ANOVA with Tukey’s *post hoc* test (****p < 0.0001, ns = not significant).

Mechanical testing highlighted key advantages of the PLA–PCL core-sheath yarn structure (Figures 3A–D). The hybrid PLA–PCL core-sheath yarn demonstrated a tenacity of 3.90 dN/tex (487.10 MPa tensile strength) and a breaking strain of 32%, outperforming pure PCL nanofibre yarns, which exhibited greater extensibility (84.72%) but significantly lower tensile strength (0.93 dN/tex or 107.26 MPa). Previous studies show that electrospun aligned PCL nanofibre yarns achieve maximum single-yarn forces of only 5.16 ± 0.04 cN (∼0.052 N/tex), underscoring their mechanical limitations (Yang Q. et al., 2022). Although bundling multiple PCL yarns increases the total force (up to 3.31 ± 0.37 N for 50 yarns), it does not fully overcome the intrinsic weakness of the material (Yang Q. et al., 2022). Melt-spun PLA filaments typically achieve tenacities between 20 and 30 cN/tex, influenced by polymer crystallinity and molecular orientation, but remain mechanically inferior compared to the hybrid system (Yang Y. et al., 2022). The integration of a PLA multifilament core with a PCL nanofibre sheath to create a core-sheath yarn results in a scalable structure suitable for engineering tubular textile scaffolds. The enhanced mechanical performance of the core-sheath yarn, compared to a PCL nanofibre yarn, is primarily attributed to the PLA multifilament core, which functions as the principal load-bearing component. In contrast, the surrounding PCL nanofibre sheath contributes to stress distribution and elasticity by mitigating localised stress concentrations and buffering tensile loads. This dual-component interaction enhances energy dissipation and ductility, consistent with observations in other multi-scale yarn architectures (Chen et al., 2022). The addition of the PCL nanofibre sheath resulted in a slight increase in strain compared to the PLA multifilament core alone, which displayed a break tenacity and strain of 3.67 dN/tex and 30.6% respectively. Although these minor mechanical improvements to the PLA multifilament core yarns were observed by the addition of PCL nanofiber sheaths, they were not statistically different. Young’s modulus values further supported these trends: the core-sheath yarn exhibited a modulus of 0.47 dN/tex, nearly identical to that of the PLA core (0.45 dN/tex), confirming that the composite’s stiffness is predominantly governed by the core. In contrast, the PCL yarn displayed a considerably lower modulus (0.064 dN/tex), reflecting its compliant nature. Overall, the PLA–PCL core-sheath yarn, with its intermediate modulus, and enhanced strength/elasticity, offers favourable properties for engineered woven scaffolds intended to leverage anisotropic mechanical behaviour. Although the tensile tests in this study were performed on individual yarns and therefore do not provide direct measurements of scaffold-level anisotropy, it is well established in textile mechanics literature that woven fabrics inherently exhibit anisotropic behaviour (Hu and Xin, 2008). This results from the directional arrangement of yarns and the structural characteristics influenced by the weave architecture. Such anisotropy is particularly relevant in the design of tubular scaffolds, where differential mechanical responses along warp and weft directions can be strategically engineered to tailor strength, compliance, and deformation characteristics to meet the specific functional demands of target tissues.

### Woven scaffolds

**FIGURE 4:**
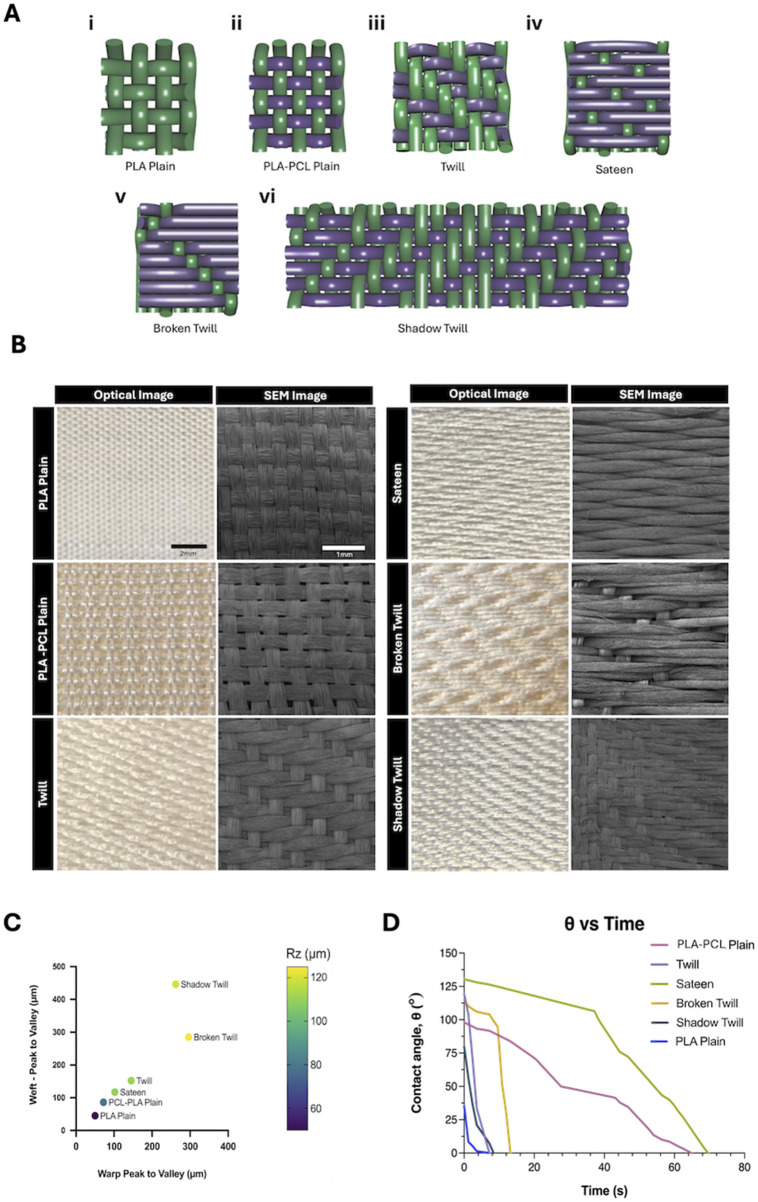
**(A)** Schematic illustrations of weave patterns and lifting plans: (i) Plain weave, (ii) PLA-PCL plain weave (iii) 2 × 2 twill, (iv) weft-focused sateen, (v) broken twill, and (vi) shadow twill. In each diagram, purple yarns represent PLA–PCL nanofibre/multifilament core-sheath yarns (weft), and green yarns represent PLA multifilament yarns (warp). **(B)** Optical and SEM images of woven patterns fabricated with PLA multifilament warp yarns and PLA–PCL core-sheath weft yarns, compared to PLA multifilament plain weave control (PLA yarns in both warp and weft). Optiacal Images: scale bar = 2 mm; SEM images: scale bar = 1 mm. **(C)** Averaged peak-to-valley height differences (warp and weft) across different weave patterns. Data points are colour-coded based on measured surface roughness (Rz, µm), illustrating the correlation between weave geometry and scaffold topography. **(D)** Contact angle measurements over time for different weave patterns, reflecting differences in wettability associated with surface structure and material composition.

Scaffolds were fabricated using an ArmPatronic dobby loom, producing five weave patterns: plain, 2 × 2 twill, sateen, broken twill, and shadow twill. PLA multifilament yarns were used as warp in all cases (20 ends/cm), while PLA or PLA–PCL yarns were used as weft. The PLA plain weave, composed entirely of PLA yarns, served as the control (Figures 4A,B). These weave patterns were selected to explore how textile structures affect scaffold mechanics and biological response. Each offers distinct advantages. Plain weave, with its high interlacing density, offers structural stability and uniform pore distribution, potentially enhancing cell infiltration. Twill and sateen weaves introduce varied float lengths and surface textures, which may impact scaffold flexibility and support directional or uniform cell growth. Broken twill and shadow twill introduce subtle 3D structures and gradient porosities that resemble the surface folds and heterogeneous textures of native tissues like the oesophagus or trachea. These features can provide mechanical support while offering topographical cues that influence cell attachment, orientation, and function. The difference in weaving patterns also demonstrated to influence surface roughness and wettability of the fabricated yarns. Average surface roughness increased with weave complexity (Figure 4C). Broken twill showed the highest roughness (73.5 ± 6.4 µm), followed by twill (65.2 ± 5.1 µm), while PLA plain had the lowest (32.6 ± 4.7 µm). Twill, broken twill, and shadow twill weaves showed higher Rz values due to longer floats and irregular interlacing (Becerir et al., 2016). Surface roughness also increased with PLA–PCL yarns compared to PLA alone, reflecting the influence of material composition (Akgun, 2015). These results demonstrate that by modulating weave pattern complexity, the degree of yarn interlacement and resulting surface topography can be precisely tuned to engineer scaffold surfaces with tailored roughness for specific biological responses (Beyene and Kumelachew, 2022). Furthermore, contact angle measurements showed significant variation across weaves where wettability correlated with surface roughness, porosity, and material composition. (Figure 4D). Increased hydrophilicity is generally favourable for cell attachment and spreading, as it promotes the adsorption of adhesive proteins like fibronectin and preserves integrin-binding motifs essential for adhesion and cytoskeletal organization (Webb et al., 1998). PLA plain exhibited a low contact angle (35°) and complete droplet absorption within 70s, indicating high hydrophilicity. PLA–PCL plain showed moderate hydrophobicity (98°) with delayed absorption over several minutes. In contrast, the sateen weave displayed pronounced hydrophobicity (131°), with minimal droplet spread and no measurable absorption over the observation period.

Constructional parameters for each weave structure, including weft densities, fabric thickness and cover factor were also measured for the different types of weaves (Table 1). Generally, fabrics with lower cover factors, such as PLA-PCL Plain Weave (43.77%), have more open area, while higher cover factors, like Broken Twill (75.37%) and Sateen (64.99%), indicate denser woven structures that enhance mechanical stability and limit permeability. Cover factor is calculated based on the yarn density and diameter in both the warp and weft directions, representing the proportion of fabric surface covered by yarns.

**TABLE 1:** Construction parameters of woven structures.

Type of weave	Weft density (picks/cm)	Warp density (picks/cm)	Weave thickness (mm)	Cover factor (%)
PLA plain	26	20	0,20	48
PLA-PCL plain	22	20	0,21	43
Twill	33	20	0,35	57
Sateen	38	20	0,45	65
Broken twill	46	20	0,51	75
Shadow twill	30	20	0,33	55

The lower tightness of the PLA–PCL plain weave compared with PLA plain, despite identical loom settings, is attributed to differences in yarn properties. The additional PCL sheath likely deformed differently under loom beat-up motion, producing slightly larger inter-yarn spacing. This variation may have influenced surface roughness and pore geometry (Figure 4C) and is considered in interpreting scaffold morphology and function.

### *In Vitro* biocompatibility evaluation of woven scaffolds

**FIGURE 5:**
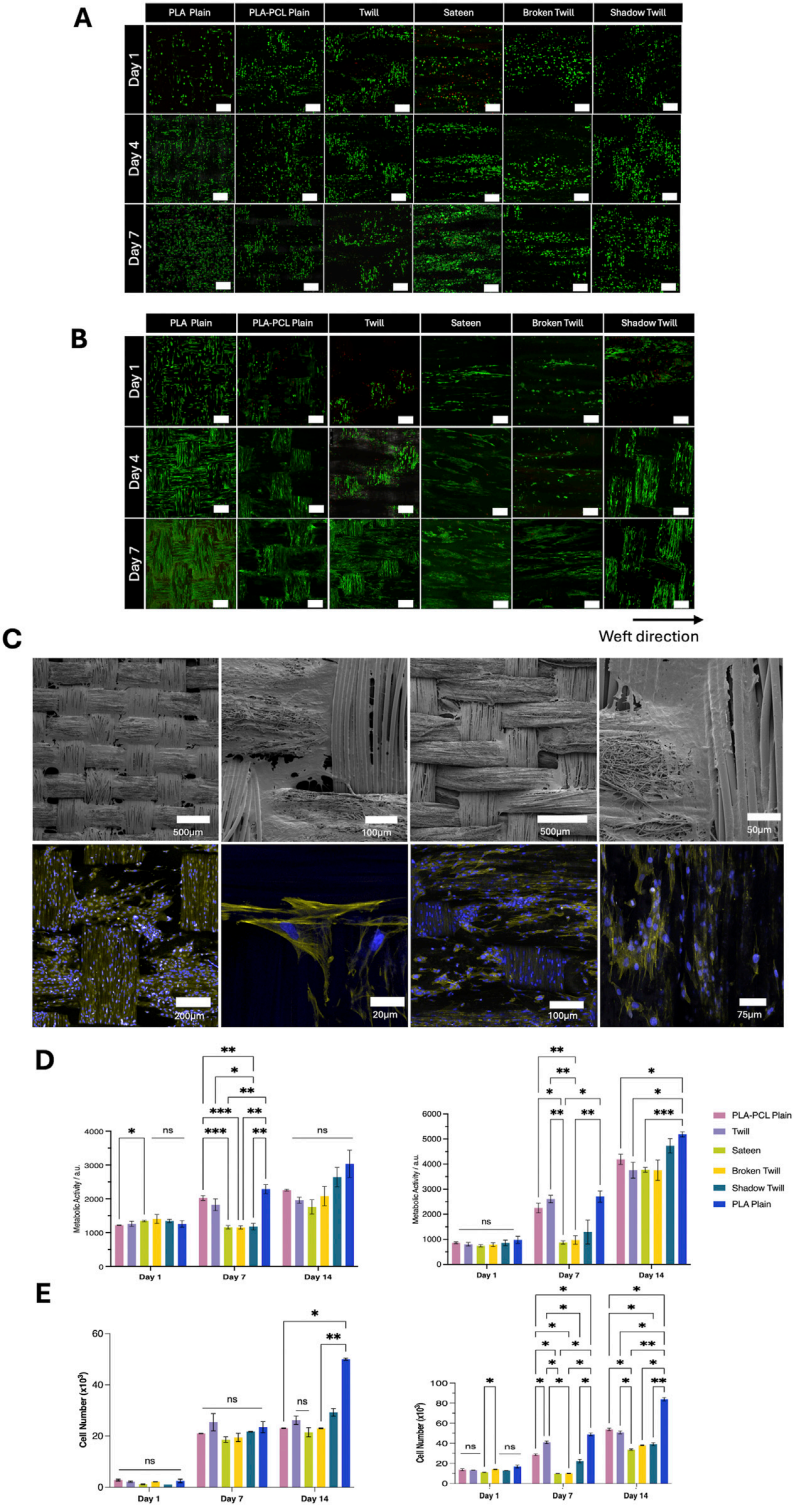
**(A)** LIVE/DEAD assay images of HUVECs cultured on woven scaffolds for day 1, 4, and 7 days. Green indicates live cells; red indicates dead cells. Scale bar = 250 µm. **(B)** LIVE/DEAD assay images of HOeSMCs cultured on woven scaffolds for day 1, 4, and 7 days. Green indicates live cells; red indicates dead cells. Scale bar = 250 µm. **(C)** SEM and confocal images of HOeSMCs on different weave patterns. (i–iv) SEM images showing cell bridging between yarns on twill and PLA–PCL weaves. (v–viii) Confocal images at day 7 showing cytoskeleton (phalloidin, yellow) and nuclei (DAPI, blue). **(D)** Metabolic activity of (i) HUVECs and (ii) HOeSMCs over 14 days, measured by resazurin reduction assay. **(E)** Cell proliferation quantified by PicoGreen dsDNA assay for (i) HUVECs and (ii) HOeSMCs. Data represent mean ± SD from two independent experiments with technical triplicates (n = 3). Statistical analysis: two-way ANOVA with Tukey’s *post hoc* test (*p < 0.05, **p < 0.005, ***p < 0.001, ns = not significant).

Biocompatibility is a key component for any scaffold used for tissue engineering and regenerative medicine applications. While PCL and PLA are widely recognized for their cytocompatibility and clinical potential (Woodruff and Hutmacher, 2010; Santoro et al., 2016), scaffold architecture can also markedly influence cell organisation. Surface topography, fibre arrangement, and pore geometry can affect cell adhesion, viability, and proliferation (Dalby et al., 2007; Anselme, 2000). Here, we evaluated how different weave structures have the capacity to support cell viability and metabolic activity across multiple cell types, recognising that both morphological and topographical cues can modulate cellular responses. The three-dimensional yarn architecture and through-thickness pores of the woven scaffolds produced complex surface topographies and multiple focal planes, which, in combination with the specific weave pattern, influenced cell organisation. Cells were frequently observed bridging yarn intersections or extending into the scaffold volume, resulting in organisation patterns that varied with the underlying weave geometry. Due to the intricacy of these architectures, conventional two-dimensional coverage measurements were not applied, as they may misrepresent cellular arrangement in such structures. Instead, qualitative fluorescence microscopy was used to evaluate cell viability and morphology, as indicators of functional organisation. LIVE/DEAD staining at days 1, 4, and 7 confirmed consistently high viability on all scaffolds (Figures 5A,B). HUVECs and HOeSMCs showed distinct attachment patterns depending on weave and fibre type. In PLA plain weaves, HUVECs and HOeSMCs exhibited a relatively uniform spatial distribution along both warp and weft yarns. In contrast, PLA–PCL plain weaves showed a more pronounced yarn-dependent pattern, with live cells preferentially located on warp yarns and fewer on the PCL nanofibre-sheathed weft. This likely reflects reduced adhesion to PCL-rich regions, where the dense nanofibrous sheath limits infiltration, compared to the open, porous PLA multifilament core, which frequently supported cell ingrowth. In more complex patterns, twill, broken twill, and shadow twill patterns showed less uniform cell arrangements. For HUVECs, which tended to be clustered in flatter regions between yarn intersections, suggesting preferential attachment to these areas over more elevated or irregular topographies. HOeSMCs, by comparison, generally aligned along the yarn axis across all weaves, with orientation changes observed at yarn intersections, reflecting adaptation to local surface geometry. Sateen weaves promoted more linear alignment along the weft, but showed reduced cell attachment during early culture, especially for HUVECs. Cell morphology and organisation were assessed using phalloidin staining of actin filaments (Figure 5C). Cell alignment and organisation were influenced by both fibre type and weave pattern. PLA multifilament yarns encouraged alignment along the filament axis and enabled infiltration between filaments (Figures 5Ci–iv), with phalloidin staining revealing continuous actin filaments spanning between neighbouring cells, indicative of close contact and suggesting the potential for network formation, while PCL nanofibre sheaths appeared to support more random, surface-level spreading, consistent with their smaller pores and lack of a dominant fibre orientation (Figure 5Cv-viii). Metabolic analysis and cell growth differences were also observed in cells seeded on different weave types. Increased metabolic activity was observed over 14 days for all cell types, with PLA plain weaves consistently showing the highest values by day 14. PLA–PCL plain and shadow twill supported moderate activity, while sateen and broken twill started with lower levels but also increased over time (Figure 5D). In SMC cultures, some weave types, particularly PLA plain, showed significantly higher metabolic activity (p < 0.05), whereas differences among HUVECs were less pronounced. Proliferation assessed via PicoGreen assay was consistent with metabolic activity as proliferation was observed to increased significantly on PLA plain (p < 0.001), with HUVECs, HOeSMCs, and HDFs showing the highest proliferation. PLA-PCL plain showed a moderate increase (p < 0.05). Twill and sateen had lower DNA content, suggesting it was less able to promote proliferation compared to other type of weaves (Figure 5E). Nonetheless, all scaffold types supported high cell viability and metabolic activity, confirming their overall cytocompatibility.

## Discussion

In this study, we systematically examined how different weave architectures in woven PLA–PCL core–sheath scaffolds influence cellular responses, focusing on spatial distribution, orientation, and network formation. While microscale parameters such as fibre diameter, surface chemistry, and material composition are well-established drivers of cell behaviour, the mesoscale arrangement defined by weave geometry represents an additional architectural variable that warrants closer attention. In this work, we examined these weave-driven patterns of cell arrangement alongside quantitative assessments of viability and metabolic activity, providing insight into how meso- and micro-scale features act together to influence scaffold performance across multiple cell types.

Clinical outcomes with tissue-engineered vascular grafts have repeatedly shown that insufficient mechanical compatibility (Yamane et al., 2017; Szafron et al., 2019), such as haemodialysis grafts and coronary bypass grafts (Lawson et al., 2016; Herrmann et al., 2019) leads to reduced patency within months. Similar issues occur where tracheal and oesophageal scaffold, where stiffness mismatches can cause collapse, granulation tissue, and impaired epithelialisation under dynamic loading (Wei et al., 2024; Luc et al., 2018; Khalid et al., 2023).

Mechanical testing confirmed the benefits of the PLA–PCL core–sheath design (Figures 3A–D). The hybrid yarn reached a tenacity of 3.90 dN/tex and a breaking strain of 32%, outperforming pure PCL nanofibre yarns in strength while retaining extensibility. Pure PCL yarns were more ductile (84.72%) but weaker (0.93 dN/tex), whereas PLA multifilament cores alone showed similar strength (3.67 dN/tex) and strain (30.6%) to the hybrid, indicating the PCL sheath mainly enhances ductility and stress distribution. This allocation of mechanical roles is consistent with the established material characteristics of PLA (stiff, high-strength) and PCL (flexible, low-strength) (Yeh et al., 2009).

Reported values for electrospun biomaterial yarns vary depending on fibre type, alignment and processing method. Aligned electrospun PCL nanofibre yarns with diameters ranging from 10 to 100 µm exhibited breaking force from approximately 1.1–5.2 cN per single yarn, with high elongation at break (140%–480%) (Yang Q. et al., 2022). When 50 of these yarns were bundled together, the total load capacity increased with yarn diameter reaching 0.44 - 3.31 N, although the increase was not linear, indicating uneven stress transfer between yarns in the bundle (Yang Q. et al., 2022). The single electrospun PCL yarn produced in this study achieved a tenacity of 0.93 dN/tex (∼9 cN/tex) confirming good mechanical performance for a single electrospun yarn compared with Yang Q. et al (2022). Electrospun PAN yarns, by contrast, demonstrate substantially higher mechanical performance, with tenacities of 7.6–9.1 cN/tex and moduli around 317 cN/tex (Wu et al., 2022). The superior strength of PAN yarns stems from their high molecular rigidity and strong intermolecular interactions, which promote efficient chain alignment and crystallinity. Multicomponent yarn systems have been investigated as a strategy to tune the mechanical properties of electrospun yarns. For example, Kruse et al. (2018) showed that blending PLA with PEG increased tensile strength from 2.5 ± 0.7 cN/tex (pure PLA) to 6.2 ± 0.5 cN/tex for a PLA 75%/PEG 25% composition (Kruse et al., 2018). A similar principle applies to the PLA–PCL core–sheath yarns in this study, where the PLA core provides the load-bearing framework and the PCL sheath introduces additional strain capacity and compliance.

In contrast to electrospun yarns, melt-spun polymer filaments can achieve substantially higher tensile properties due to their greater molecular orientation and crystallinity. For example, Bauer et al. (2022) reported PCL monofilaments with tensile strengths up to 69 cN/tex, obtained through drawing-induced macromolecular alignment and strain-induced crystallisation. Similarly, textile-grade multifilaments fabricated from PLA, PLCL, PDS, and PLAGA for ligament scaffold applications exhibited tenacities in the range of 30–47 cN/tex (Hahn et al., 2019; Cooper et al., 2011). These values exceed those typically achieved in electrospun systems. Although the hybrid yarns in this study do not reach the strength of highly oriented melt-spun fibres, their tenacity falls within the range reported for textile-grade degradable filaments (Hahn et al., 2019; Cooper et al., 2011) and is substantially higher than that of typical electrospun yarns.

For tubular scaffold design, these yarn-level properties have direct implications. In woven architectures, yarn tensile properties can influence fabric tensile modulus, burst pressure resistance, and compliance. The high tenacity and moderate extensibility of PLA–PCL core–sheath yarns could potentially be advantageous for withstanding physiological pressures while avoiding excessive stiffness that can cause compliance mismatch for tubular graft applications. Compared to native vascular tissues like the ascending aorta, which typically exhibit ultimate tensile strengths of ∼0.8–4.1 MPa and strain at failure between 20% and 60% depending on the region (Ferrara et al., 2016; Azadani et al., 2012). The yarn properties reported here provide a mechanical baseline capable of matching or exceeding native vessel strength (487.10 MPa) which can significantly increase when integrated into a tight plain weave fabric. Furthermore, the rigidity of conventional grafts knitted out of Dacron® or ePTFE has often been associated with compliance mismatch complications that lead to graft failure such as false aneurysm, intimal hyperplasia and anastomotic stenosis (Lucereau et al., 2020 and Fang et al., 2021). The addition of our PCL nanofibre sheaths not only showed to improve strength of our yarns but also increase their ability to deform/extend. Therefore, such modifications at the yarn level can potentially enhance the flexibility of grafts and reduce complications associated with their rigidity. This positions the scaffolds produced via our method within a functional range for medium-to large-diameter vascular grafts, where controlled compliance are critical to haemodynamic compatibility and long-term graft patency. Moreover, the modularity of woven construction allows adjustment of pick density, weave pattern, and yarn composition to fine-tune scaffold mechanics for specific anatomical locations.

The incorporation of a compliant PCL nanofibrous sheath around a stiffer PLA multifilament core maintained high tensile strength (3.90 dN/tex) while imparting a modest increase in extensibility (32% compared with 30.6% for PLA alone). This core–sheath configuration provides the ability to independently tailor mechanical and surface properties, enabling the adjustment of scaffold compliance to fall within the physiological range of target tissues. Such tunability is advantageous for designing woven architectures intended for dynamic loading environments.

Processing parameters, including twist uniformity and sheath coverage, were critical for ensuring consistent yarn performance. Excessive twist could disrupt nanofibre alignment and introduce local stress concentrations (Bazbouz and Stylios, 2012). Although increasing the twist or modifying the sheath material may improve abrasion resistance by enhancing inter-fibre friction, such adjustments could adversely affect yarn uniformity and complicate textile processing (Zhou et al., 2010). While scaffold-level mechanical validation remains necessary, these findings establish a clear pathway for translating yarn-level design choices into woven architectures optimised for specific tissue engineering applications.

Our method demonstrates that woven scaffolds fabricated from PCL-PLA core-sheath nanofibre yarns can be engineered with precisely controlled topographies, porosities, and surface characteristics by varying the weave architecture. These controlled meso- and micro-scale features directly influenced scaffold morphology and cellular responses, with distinct weave patterns significantly affecting cell alignment and cytocompatibility of HOeSMCs- and HUVECS-laden scaffolds This relationship between architecture and biological performance was evident when comparing specific weave types. Weave patterns, through their control of yarn interlacement and surface topography, provided physical cues that guided cell behaviour. As shown in Figure 5, each weave architecture produced distinct morphological features, including fibre diameter, pore geometry, and surface roughness, which in turn shaped cellular organisation. PLA multifilament and PLA–PCL plain weaves exhibited highly uniform structures with minimal height variations, which correlated with consistently high cell viability (LIVE/DEAD) and DNA content.

Phalloidin staining confirmed uniform cytoskeletal organisation and directional spreading along warp and weft yarns. In contrast, complex patterns like twill and broken twill introduced irregular topographies that disrupted alignment and promoted multilayered or clustered cell distributions.

While weave architecture governs spatial characteristics of pore distribution and surface roughness, material composition introduced an additional layer of control. The PLA multifilament core and PCL nanofibrous sheath offered complementary microenvironments that further refined cellular responses. Consistent with previous studies (Flavia et al., 2022; Cai et al., 2023)., PLA multifilament have shown to enable cell infiltration and alignment along individual fibres, consistent with their open pore structure and larger pore sizes. This architecture is advantageous for applications requiring robust 3D cellular integration within the scaffold (Flavia et al., 2022; Cai et al., 2023). In contrast, the PCL nanofibre scaffolds, characterised by low porosity and small pore sizes, create a dense surface environment that facilitates morphology consistent with cohesive monolayer formation and promotes cohesive monolayer development by endothelial cells (Chernonosova and Laktionov, 2022; Jain et al., 2023). In our study, PLA-PCL multifilament/nanofibrous yarns combined these attributes, producing scaffolds that balanced infiltration with surface coverage. Preliminary cytocompatibility with 3T3 fibroblasts confirmed strong adhesion and sustained growth on both components. Integrating multifilament and nanofibrous architectures within a single yarn enables layer-specific microenvironments, offering a versatile strategy for engineering multilayered tubular tissues (Ramakrishna, 2001; Blakeney et al., 2011; Pham et al., 2006). The structural design of the woven scaffolds influenced cellular organisation by providing topographical cues through the orientation and interlacement of the yarns, which affected cytoskeletal arrangement and cell alignment. Plain weave architectures, characterised by uniform pore geometry and low surface roughness, supported more consistent cell orientation and viability, whereas more complex patterns such as twill and broken twill introduced irregular topographies that reduced overall alignment. These findings suggest that the geometric configuration of the weave can contribute to guiding cellular behaviour in woven tissue-engineering scaffolds.

Recent melt-electrowriting (MEW) studies provide an alternative microfabrication route for tubular scaffolds, demonstrating precise filament alignment and reporting scaffold-level compliance and burst measurements (Bartolf-Kopp et al., 2024). In contrast, the present work uses a textile strategy utilising core-sheath nanofibre yarns interlaced by weaving to build hierarchical, yarn- and weave-defined architectures that operate at a different structural length scale from MEW’s printed microfilaments.

Our findings show that scaffold performance is jointly dictated by fibre-level morphology and weave-level geometry, and that tuning both scales in of application-specific architectures that better replicate native tissue organisation. These findings underscore that both nano- and meso-scale architectural control are critical not only for supporting cell viability and organisation but also for designing scaffolds capable of replicating the functional and structural complexity of native tubular tissues.

## Conclusion

The development of scaffolds for tubular soft tissues requires careful consideration of design parameters, material properties, and mechanical performance to meet their complex biological and functional demands. Here, we present a scalable weaving approach combining PLA multifilament cores with electrospun PCL nanofibre sheaths to fabricate core-sheath yarns, which are then woven into tubular scaffolds with tuneable architectures and topographies. PCL added to plain PLA core yarns demonstrated modification of morphological features while different weaves had significant influence in cell function and alignment. Importantly, this study evaluated yarn-level mechanics only, tubular compliance, burst pressure, and scaffold-level anisotropy were not measured and are not claimed here. Our method offers a scalable and precise solution to other methods, such as manual weaving or conventional electrospinning, both of which are limited by scalability and dimensional stability. Furthermore, the ability to easily tuneable weave patterns to change scaffold topography provides cell specific cues that can influence cell function and alignment. Future work will focus on evaluating the mechanical performance of tubular scaffolds under realistic physiological conditions to test important parameters such as burst pressure and tensile stress, optimising warp/weft densities to better control morphological characteristics such as pore dimensions and transitioning to a fully automated fabrication process. This automation, enabling precise control of weft insertion and beat-up, will enhance reproducibility, scalability, and potentially improve nutrient exchange in tissue engineering or clinical applications.

## Data Availability

The raw data supporting the conclusions of this article will be made available by the authors, without undue reservation.
